# Going above and beyond for implementation: the development and validity testing of the Implementation Citizenship Behavior Scale (ICBS)

**DOI:** 10.1186/s13012-015-0255-8

**Published:** 2015-05-07

**Authors:** Mark G Ehrhart, Gregory A Aarons, Lauren R Farahnak

**Affiliations:** Department of Psychology, San Diego State University, San Diego, CA USA; Department of Psychiatry, University of California, San Diego, La Jolla, CA USA; Child and Adolescent Services Research Center, San Diego, CA USA

**Keywords:** Implementation citizenship behavior, Organizational citizenship behavior, Evidence-based practice, Organizational context, Measurement, Mental health

## Abstract

**Background:**

In line with recent research on the role of the inner context of organizations in implementation effectiveness, this study extends research on organizational citizenship behavior (OCB) to the domain of evidence-based practice (EBP) implementation. OCB encompasses those behaviors that go beyond what is required for a given job that contribute to greater organizational effectiveness. The goal of this study was to develop and test a measure of implementation citizenship behavior (ICB) or those behaviors that employees perform that go above and beyond what is required in order to support EBP implementation.

**Methods:**

The primary participants were 68 supervisors from ten mental health agencies throughout California. Items measuring ICB were developed based on past research on OCB and in consultation with experts on EBP implementation in mental health settings. Supervisors rated 357 of their subordinates on ICB and implementation success. In addition, 292 of the subordinates provided data on self-rated performance, attitudes towards EBPs, work experience, and full-time status. The supervisor sample was randomly split, with half used for exploratory factor analyses and the other half for confirmatory factor analyses. The entire sample of supervisors and subordinates was utilized for analyses assessing the reliability and construct validity of the measure.

**Results:**

Exploratory factor analyses supported the proposed two-factor structure of the Implementation Citizenship Behavior Scale (ICBS): (1) Helping Others and (2) Keeping Informed. Confirmatory factor analyses with the other half of the sample supported the factor structure. Additional analyses supported the reliability and construct validity for the ICBS.

**Conclusions:**

The ICBS is a pragmatic brief measure (six items) that captures critical behaviors employees perform to go above and beyond the call of duty to support EBP implementation, including helping their fellow employees on implementation-related activities and keeping informed about issues related to EBP and implementation efforts. The ICBS can be used by researchers to better understand the outcomes of improved organizational support for implementation (i.e., implementation climate) and the proximal predictors of implementation effectiveness. The ICBS can also provide insight for organizations, practitioners, and managers by focusing on key employee behaviors that should increase the probability of implementation success.

**Electronic supplementary material:**

The online version of this article (doi:10.1186/s13012-015-0255-8) contains supplementary material, which is available to authorized users.

## Introduction

Organizational citizenship behavior (OCB) has been defined as “individual behavior that is discretionary, not directly or explicitly recognized by the formal reward system, and in the aggregate promotes the efficient and effective functioning of the organization” (p. 3) [1]. OCB is related to a number of indicators of individual-level and unit-level effectiveness [2-4]. For instance, OCB is positively associated with managerial performance evaluations [5,6], actual performance [7,8], sales team effectiveness [9], production quality [10], and health care outcomes [11-14]. In addition, OCB has been shown to be negatively related to turnover and intentions to quit [4].

Recently, OCB research has moved to take on a specific focus, such as customer-focused OCB and safety OCB, and to demonstrate relationships with outcomes of the same focus. For example, in the service literature, bank branch-level extra-role customer service behaviors (i.e., customer-focused OCB) have been shown to be positively related to customer satisfaction [15,16]. There has also been an increased interest in research on safety-specific OCB and its influence on safety-specific organizational outcomes. Griffin and Neal [17] argued that safety-focused OCB was a distinct form of safety performance that captured employees’ voluntary participation in safety activities that “help to develop an environment that supports safety” (p. 349). Safety-focused citizenshipbehavior includes such activities as voluntarily participating in safety meetings [17], promoting safety programs within the organization [18], and raising safety concerns [19].

This paper extends research on focused OCB to evidence-based practice (EBP) implementation. In health and mental health settings, there has been increasing attention to the development and use of EBPs with rigorous empirical support for their efficacy and effectiveness that also allow for clinical expertise and patient preference [20]. In the United States, the use of such EBPs has been more commonly tied to federal and state funding as policy makers work to ensure that their financial support is being used in ways that will maximize the likelihood for more positive patient outcomes and public health impact [21,22]. One of the biggest challenges to the widespread application of EBPs is effective implementation, and one of the biggest challenges to effective implementation is the organizational context in which the implementation takes place [23,24]. Implementation researchers have developed a number of measures intended to capture the issues specifically relevant to implementation in health and allied health care settings (e.g., implementation leadership [25], implementation climate [26,27], and molar climate, or general organizational social context [28]). However, this research has not been extended to the actual focused behaviors performed by employees during the implementation process, specifically in the form of implementation citizenship behavior (ICB).

The goal of the present study was to address this gap in the literature through the development of an ICB scale (ICBS) designed to measure behaviors as they specifically relate to EBP implementation and associated outcomes. We define ICB as the discretionary behavior employees perform to support EBP implementation. Examples of such behaviors may include demonstrating a commitment to EBP, supporting the use and integration of EBP into clinical care, and holding others in the organization or team to the highest standards of EBP. There is very little literature on the behaviors that direct service health care providers perform to facilitate implementation, as much of the focus is on formal leaders [23], opinion leaders [29], champions [30], or change agents [31]. One exception can be found in Damschroder et al.’s [32] Consolidated Framework for Implementation Research (CFIR). One of the major domains in the CFIR is characteristics of individuals, which includes a set of constructs that includes OCB labeled as “individual identification with the organization”. In describing these constructs, Damschroder et al. [32] note, “These measures have been studied very little in healthcare, but may be especially important when evaluating the influence of implementation leaders… on implementation efforts” (p. 10). Thus, ICBs may play an important role for demonstrating employee engagement in the implementation process and for exhibiting positive peer influence towards EBP implementation and use.

We developed the ICBS based on past research on focused OCB, capturing two dimensions (helping behaviors and keeping informed) that were considered to be the most relevant for EBP implementation and that captured ICB targeted towards other individuals and ICB towards the organization as a whole, in line with how this distinction has been made in the OCB literature [33]. We evaluated the ICB’s scale characteristics through an examination of factor structure and internal consistency reliabilities. In addition, we examined evidence for the construct validity of the scale through the inclusion of a number of additional measures. Specifically, we included measures to support the convergent evidence of validity via significant correlations of varying strength. Based on past OCB research suggesting that supervisors take into account their subordinates’ OCBs when evaluating their performance [4] and because of frameworks like the CFIR [32] that identify implementation-oriented OCBs as critical for implementation effectiveness, we expected supervisor ratings of ICB to be strongly correlated with supervisors’ perceptions of the employees’ implementation success, as well as moderately correlated with employees’ ratings of their own implementation success. We expected employee attitudes about EBPs to have small-to-moderate correlations with supervisor ratings of ICB, in line with the literature on job attitudes and OCB [34]. Finally, we expected employee experience in mental health and full-time status to have weak-to-moderate relationships with ICB. Past research indicates that individuals with more expertise and who are more accessible have more opportunities to help their coworkers [35]; in addition, individuals with more experience and who have full-time status should have more resources available to them to keep informed on issues related to EBPs.

In summary, the goal of this study was to develop a scale measuring ICB directed towards other individuals (helping) and towards the organization as a whole (keeping informed) that was pragmatic and brief to allow for use of the scale in both research and applied settings.

## Method

### Item generation

Throughout the item generation process, we received input from subject matter experts in EBP implementation in mental health settings to ensure the content of the items was appropriate for this setting. The subject matter experts included a mental health program leader with extensive experience working in public sector mental health programs and particularly with managing the implementation of EBPs, and an EBP trainer and Community Development Team consultant from the California Institute for Mental Health [36] with broad expertise in the challenges of EBP implementation and deep knowledge of implementing multiple EBPs across numerous community-based organizations, as well as members of the research team with expertise in organizational citizenship behaviors and EBP implementation. The ICBS items were adapted from a measure developed by Hofmann, Morgeson, and Gerras [37] that assesses citizenship behavior related to improving safety. Their measure included 27 items across six scales (helping, voice, stewardship, whistle-blowing, civic virtue/keeping informed, initiating safety-related change). OCB is often divided into two categories: behaviors that are focused on the individual (OCB-I) and behaviors that are focused on the organization as a whole (OCB-O). In line with this distinction, for the ICBS, the authors chose one OCB-I dimension (helping) and one OCB-O dimension (keeping informed), which were determined to be the most relevant to EBP implementation based on subject matter expert input. The items were adapted through consultation with the subject matter experts to enhance fit with EBP implementation. Once the initial items had been drafted, the scale was also reviewed by four mental health program managers for additional feedback regarding face validity and content validity. The items were then finalized, with any questions being resolved by the subject matter expert consultants. The final set of ten items represented two potential content domains of implementation citizenship behavior: (1) Helping Others and (2) Keeping Informed.

### Participants and procedure

Participants were mental health supervisors and their supervisees from ten mental health agencies throughout California. At the time of data collection (2012–14), all service providers were implementing or using one or more EBPs with clients. Of the 73 eligible supervisors, 68 supervisors of distinct work groups agreed to participate (response rate = 93.15%). Supervisors provided ratings of implementation citizenship behavior for a total of 357 providers (average of 5.25 providers per supervisor; range = 1–14). Of these providers, 292 also filled out the employee survey that provided data for the construct validity analyses. Demographic information for supervisors and providers is provided in Table 1. Agencies were recruited through contact with agency executives, and data were collected using online surveys or paper-and-pencil surveys. In all cases, the supervisor completed his/her survey in a separate location from his/her subordinates to ease any concerns from participants about the confidentiality of their responses. Incentives in the form of gift cards to a large online retailer were provided to both the providers and the supervisors for completing the survey. Service providers received $15 incentives and supervisors, who had longer surveys to complete, received $30 incentives. The study was approved by the appropriate institutional review boards, all participants provided consent to participate, participation was voluntary, and participants could decline or withdraw from the study at any time without any negative consequences.

**Table 1:** **Demographics of supervisors and service providers**

**Supervisors (** ***N*** **= 68)**		**Service providers (** ***N*** **= 292)**	
Race		Race	
Caucasian	66.7%	Caucasian	46.5%
African-American	4.8%	African-American	14.9%
Asian-American	11.1%	Asian-American	7.8%
Native American	3.2%	Native American	0.4%
Other	14.2%	Other	30.5%
Ethnicity		Ethnicity	
Hispanic	15.9%	Hispanic	41.5%
Non-Hispanic	84.1%	Non-Hispanic	58.5%
Education		Education	
No college	1.6%	No college	2.4%
Some college	7.9%	Some college	5.8%
College degree	1.6%	College degree	25.3%
Master’s degree	81.0%	Master’s degree	64.4%
Ph.D. or M.D.	7.9%	Ph.D. or M.D.	2.1%
Age		Age	
Mean (SD)	42.76 years	Mean (SD)	38.09 years
	(12.45)		(10.58)
Tenure with agency		Tenure with agency	
Mean (SD)	6.39 years	Mean (SD)	3.58 years
	(4.78)		(2.96)
Tenure in mental health		Tenure in mental health	
Mean (SD)	13.79 years	Mean (SD)	6.61 years
	(7.94)		(5.30)
Gender		Gender	
Female	77.8%	Female	78.1%
Male	22.2%	Male	21.9%

### Measures

#### Implementation citizenship behavior scale

The ICBS was originally developed as a part of an NIMH measure development grant to assess the extent to which employees exceed their expected job tasks to support the implementation of EBPs. Ten items were developed and evaluated based on the development process described above. In this measure, supervisors assessed each of their follower’s implementation citizenship behavior. All ICBS items were scored on a five-point, 0 (“not at all”) to 4 (“frequently, if not always”), scale.

### Construct validity measures

Implementation success was assessed through employee self-report and supervisor ratings. Supervisors assessed each of their follower’s preparation, competence, fidelity, and overall success in EBP on a five-point, 0 (“not at all”) to 4 (“to a very great extent”), scale. This measure had good internal consistency (*α* = .97, 4 items). Self-rated performance was assessed using a single item that asked employees to rate the extent to which they use EBP with fidelity on a five-point, 0 (“not at all”) to 4 (“to a very great extent”), scale. Attitudes towards EBP were measured using the Evidence-Based Practice Attitude Scale (EBPAS) [38,39]. The EBPAS includes 15 items that assess provider attitudes towards adoption of and EBP in mental health, social service, and alcohol/drug settings. The EBPAS consists of a total scale score (*α* = .91) and four lower-order factors/subscales: Requirements (*α* = .96, 3 items), Appeal (*α* = .79, 4 items), Openness (*α* = .80, 4 items), and Divergence (*α* = .68, 4 items). Items are scored on a five-point, 0 (“not at all”) to 4 (“to a very great extent”), scale. Finally, mental health experience was measured based on an item asking about providers’ years of experience in mental health, and full-time status was taken from a question asking providers if they were employed full-time or part-time.

### Statistical analyses

The sample was randomly split so that half could be utilized for the exploratory factor analysis (EFA) and half for the confirmatory factor analysis (CFA). Because of dependencies in the individual-level data due to supervisors rating multiple individuals (i.e., for providers that report directly to the same supervisor), we split the sample at the supervisor level within organizations. This resulted in a sample of 178 providers reporting to 34 supervisors for the EFA and 179 providers reporting to 34 supervisors for the CFA.

Mplus statistical software [40] was used for both the EFA and CFA analyses. The EFAs accounted for the nested data structure (because supervisors rated multiple individuals) and allowed for correlated factors (because past OCB research has shown the various dimensions to be related to each other). Specifically, we utilized maximum likelihood estimation with robust standard errors (MLR), using the clustering command to account for the nested data and Promax oblique rotation to allow for correlated factors. The number of factors was determined based on multiple criteria including the variance accounted for by the solution, the variance accounted for by each individual factor, the interpretability of the factors, and parallel analysis [41-43]. The initial criteria for item inclusion were primary loadings above 0.40 and cross-loadings below 0.30 [44]. Because we aimed to develop a brief and pragmatic measure to maximize its usefulness in both research and practice, we subsequently evaluated items based on their relative loadings on a given factor, whether they directly assessed the factor’s content (i.e., helping or keeping informed) and whether they would be applicable and understandable across a broad range of participants. Parallel analysis was based on estimation of 1,000 random data matrices values that correspond to the 95th percentile of the distribution of random data eigenvalues. The random values were then compared with derived eigenvalues to determine whether the parallel analysis supported the number of factors identified in the EFA [42].

Once the factor structure was determined based on the EFA, it was tested in the other half of the sample using CFA. The CFA was also conducted in Mplus accounting for the nested data structure using maximum likelihood estimation with robust standard errors (MLR), which appropriately adjusts standard errors and chi-square values. Missing data were imputed through full information maximum likelihood (FIML) estimation (note that missing data was minimal and varied from zero to four missing cases across the items in the CFA). Model fit was assessed using several empirically supported indices: the comparative fit index (CFI), the Tucker-Lewis index (TLI), the root mean square error of approximation (RMSEA), and the standardized root mean square residual (SRMR). CFI and TLI values greater than 0.90, RMSEA values less than 0.10, and SRMR values less than 0.08 indicate acceptable model fit [45]. Type two error rates tend to be low when multiple fit indices are used in studies where sample sizes are large and non-normality is limited, as in the present study [46].

We tested the internal consistency reliability of the final scales (total scale and subscales) using Cronbach’s alpha. Finally, construct validity was assessed by computing correlations between the ICBS measure (overall scale and subscales) and the construct validity measures.

## Results

### Exploratory factor analysis

An iterative EFA process was used applying the criteria described above. Based on the initial factor solution, one item was removed after the first iteration based on statistical criteria (cross-loading above 0.30). In the second and final iteration, one additional item was removed because of overlapping content with another item with a higher factor loading and two items were removed because of language that may be unfamiliar or vague to participants (“implementation activities”). These three items also had the lowest factor loadings. In total, four items were removed, resulting in a final scale of six items loading on two factors.

The variance explained by the final EFA solution was 87.94%, and the two factors individually accounted for 72.90% and 15.04% of the variance, respectively. Eigenvalues were 4.38 for the first factor and 0.90 for the second factor; although the eigenvalue for the second factor did not meet the traditional cutoff of 1.00, we retained the two-factor solution based on our theoretical model and suggestions in the literature to not use 1.00 as a strict cutoff for eigenvalues [42]. In addition, the parallel analysis indicated that a two-factor solution best represented the data. Using the rotated solution for interpretation, three items loaded onto each factor and the items had high factor loadings (see Table 2). Both factors were consistent with the original proposed dimensions (Helping Others and Keeping Informed). The correlation between these factors (*r* = 0.62) suggests that the subscales are correlated, but not overlapping.

**Table 2:** **EFA and CFA results for the implementation citizenship behavior scale**

	**EFA factor loadings**		**CFA factor loadings**	**Item-total correlations**
**ICBS items and subscales**	**1**	**2**		
1. Helping Others				
Responsibilities related to EBP implementation	**0.91**	−0.01	0.86	0.84
Make sure they implement EBP properly	**0.86**	0.10	0.92	0.86
Helping teach EBP implementation procedures	**0.83**	0.11	0.89	0.84
2. Keeping Informed				
Agency communication related to EBP	−0.03	**0.91**	0.79	0.79
Latest news regarding EBP	0.08	**0.86**	0.88	0.83
Changes in EBP policies and procedures	0.15	**0.80**	0.92	0.84

Bold font for EFA factor loadings indicates the scale on which the items load. *EFA* exploratory factor analysis, *CFA* confirmatory factor analysis, *ICBS* Implementation Citizenship Behavior Scale, *EBP* evidence-based practice.

### Confirmatory factor analysis

Using the other half of the randomly split sample, the CFA tested the two-factor model with a higher-order factor representing ICB. This model demonstrated excellent fit as indicated by multiple fit indicators (CFI = 1.00; TLI = 1.02; RMSEA = 0.00; SRMR = 0.02). As shown in Table 2, the standardized factor loadings ranged from 0.79 to 0.92 and all factor loadings were statistically significant (*p*’s < .001). In addition, the loadings of Helping Others and Keeping Informed on the ICB second-order factor were 0.84 and 0.96, respectively. Based on these findings, we accepted the two-factor model without additional modification. The ICBS and scoring instructions can be found in Additional file 1 and Additional file 2, respectively, or may be obtained from GAA.

### Scale reliability statistics

Table 3 shows ICBS total scale and item means and SDs and the scale reliabilities using the full sample (i.e., participants in both the EFA and CFA sample). Internal consistencies for the Helping Others (*α* = 0.93) and Keeping Informed (*α* = 0.91) subscales were excellent, as it was for the overall scale (*α* = 0.93). Item analyses indicated that item-total correlations for items and their subscales were high, ranging from 0.79–0.86 (see Table 2).

**Table 3:** **Correlations among the study variables**

	***M***	**SD**	**1**	**2**	**3**	**4**	**5**	**6**	**7**	**8**	**9**	**10**	**11**
1. ICBS	2.06	1.08	(.93)										
2. ICBS: Helping Others	1.97	1.23	0.92**	(.93)									
3. ICBS: Keeping Informed	2.16	1.13	0.91**	0.69**	(.91)								
4. Implementation success (employee rated)	2.44	1.11	0.30**	0.33**	0.22**	-							
5. Implementation success (supervisor-rated)	2.38	1.16	0.81*	0.77**	0.72**	0.42**	(.97)						
6. EBPAS	2.80	.51	0.14*	0.12*	0.12*	0.30**	0.16**	(.91)					
7. EBPAS: Requirements	2.76	1.02	0.06	0.05	0.07	0.27**	0.08	0.76**	(.96)				
8. EBPAS: Appeal	2.91	.69	0.14*	0.14*	0.11	0.21**	0.14*	0.72**	0.36**	(.79)			
9. EBPAS: Openness	2.82	.70	0.12*	0.14*	0.08	0.32**	0.20**	0.63**	0.25**	0.51**	(.80)		
10. EBPAS: Divergence	1.31	.73	−0.04	−0.01	−0.06	0.04	−0.02	−0.43**	−0.11	−0.05	0.04	(.68)	
11. Experience	79.30	63.55	0.11	0.13*	0.07	0.12	0.04	0.08	0.10	0.10	0.11	−0.10	-
12. Full-time status	1.04	0.21	−0.15**	−0.20*	−0.08	−0.12	−0.17**	−0.03	−0.02	−0.00	−0.03	−0.04	−0.01

Note: *N* = 357 for correlations with supervisor-rated implementation success. Due to missing data on some items/scales, sample size for correlations with the data from employees ranges from 220–289. Experience was measured in months. *ICBS* implementation citizenship behavior scale, *EBPAS* evidence-based practice attitude scale. **p < 0.01; *p < 0.05.

### Construct validity analyses

To assess construct validity, we examined correlations for the ICBS total score and its two subscales with supervisor-rated and provider-rated implementation success, provider-rated attitudes towards EBP, experience, and full-time status. The results are shown in Table 3. As predicted, the ICBS total score was strongly correlated with implementation success (*r* = 0.81, *p* < 0.01), as were both subscales (Helping Others: *r* = 0.77, *p* < 0.01); Keeping Informed: *r* = 0.72, *p* < 0.01). In addition, there was a moderate correlation with provider-rated implementation success for the total score (*r* = 0.30, *p* < 0.01) and the Helping Others dimension (*r* = 0.33, *p* < 0.01) and a slightly weaker but still significant correlation for the Keeping Informed dimension (*r* = 0.22, *p* < 0.01). The correlations between the total score for EBP attitudes and the ICBS measure and its dimensions were all significant (*r’s* = 0.12-14, *p* < 0.05), which were generally in line with expectations but slightly weaker than past research on the relationship between attitudes and OCB [34]. Only two of the EBPAS dimensions were significantly correlated with the ICBS total score and Helping Others dimension (Appeal and Openness), and none were significantly correlated with the ICBS Keeping Informed dimensions. Finally, experience in mental health was significantly correlated with the ICBS Helping Others dimension (*r* = 0.13, *p* < 0.05), and full-time status was significantly correlated with both the ICBS total score (*r* = 0.15, *p* < 0.05) and the Helping Others subscale (*r* = 0.20, *p* < 0.05). Neither experience nor full-time status was significantly correlated with the ICBS Keeping Informed subscale. Overall, these results were in line with the expected pattern of results and support the construct validity of the ICBS.

## Discussion

The purpose of this study was to develop a brief, practical, reliable, and valid measure of implementation-focused citizenship behavior. Drawing from research on extant literature on OCB [1,33,37] and with the input of subject matter experts in the domain of EBP implementation, we developed items capturing citizenship behaviors targeted towards other individuals (i.e., helping others) and towards the organization as a whole (i.e., keeping informed). Our analytic approach allowed us to reduce the number of items tapping these dimensions to three items each for a total of six items. This is consistent with emerging measurement work in implementation science that seeks to develop robust and pragmatic measures that can be efficiently used for research and for implementation process support [25-27]. Confirmatory factor analyses provided strong support for the overall structure of the scale, including strong factor loadings and overall fit. In line with these results, the internal consistency reliabilities for the overall scale and its two dimensions were strong.

There was also strong support for the construct validity of the ICBS. Clearly, supervisors see a close relationship with their employees’ implementation success and ICB, with correlations in the .72 to .81 range. Perhaps more interesting were the correlations between the providers’ ratings of their own implementation fidelity and the supervisor ratings of ICB, as these provide additional support for the association between implementation success and ICB using two different sources. The findings for employee attitudes were in line with expectations, although perhaps weaker than expected. That being said, one of the two scales that was not related to ICB was the Requirements subscale, which asks about willingness to adopt an EBP if required to do so and thus is not in line with the idea of OCB as behavior that goes beyond typical requirements. In that regard, this finding does make sense. In addition, ICB was not related to the Divergence subscale, which captures perceived divergence between EBPs and usual care. This is in contrast to the two subscales that were related to ICB, Appeal, and Openness, which capture positive attitudes towards EBPs. Thus, ICB was most closely related to positive attitudes about EBP. Nevertheless, the strength of the significant correlations (.12–.14) was lower than what Organ and Ryan [34] found for attitudes predicting OCB (.19–.23 uncorrected). Future research should further investigate possible moderators that may affect the strength of this relationship. Finally, the ICBS was related to both experience (with Helping Others) and full-time status (with the total score and Helping Others subscale). Although these relationships were tested for measure validation purposes in this manuscript, they do provide a basis for future research to expand on to further clarify the mechanisms through which those with more experience and who work full-time have more opportunities to help their coworkers with implementation-related activities.

One consistent finding throughout the construct validity results was that the correlations were typically stronger for the Helping Others subscale than for the Keeping Informed subscale. A possible explanation for this finding is that supervisors are less aware of employees’ actions to stay informed about EBP, whereas employees’ helping their coworkers with implementation are more public and thus supervisor ratings are more accurate with regard to that dimension. It may also be the case that the variables included in the construct validity analyses tapped issues that were more relevant for Helping Others than for Keeping Informed. Future research should expand the nomological network of these dimensions to better understand their unique correlates. One such variable is the sex of the participant, as helping behavior is traditionally more associated with females than males [47]. Post hoc exploratory analyses revealed no such difference in this sample; however, women were rated higher in keeping informed than men (*M* (women) = 2.28, *M* (men) = 1.86, t (290) = 2.72, *p* < .01). Future research should explore the role of employees’ sex and gender in implementation citizenship behavior. In addition, future research should include providers’ self-ratings of their ICB to see how this affects the pattern of relationships with related constructs, as well as providers’ role definitions, as past research has shown that how broadly employees define the behaviors included in their work role has important implications for their likelihood of performing citizenship behavior [48,49].

One potential limitation of the present study is that it utilized a sample in the allied health care setting of mental health agencies. Future research should explore the ICBS’s utility in other settings in which implementation of EBP is a strategic imperative. Because our study focused on organizations that used multiple forms of EBP, we generalized the item wording when describing EBP. Even if the wording were adapted to fit a specific EBP, we would anticipate that the scale would remain meaningful and empirically supported; however, more research is needed to see if a focus on specific EBPs would impact the strength of the correlations. Another potential limitation is the sample size for the EFA, which consisted of 178 providers rated by 34 supervisors. However, past research on the appropriate sample size for factor analysis indicates that such sample sizes may be appropriate when communalities are high and the factors are overdetermined [50]. Because our findings met these criteria and were also validated in the CFA, we concluded that the sample size was adequate for a stable solution. Finally, the importance of ICB is its impact on implementation and implementation-related outcomes; thus, future research should include a broader range of effectiveness variables, particularly those directly related client/patient outcomes.

## Conclusions

The current study builds on past OCB research by extending it to a specific or focused form of OCB (i.e., implementation citizenship). Although past research on the organizational context for EBP implementation has provided insight on such issues as leadership [25], climate [24,26,27], and provider attitudes [38], the ICBS provides insight into clinician behavioral outcomes that are likely influenced by these antecedent variables and that subsequently are likely tied to implementation effectiveness. From a practical perspective, the ICBS allows organizations to understand and measure how behaviors specific to implementation directly impact implementation outcomes such as efficiency and effectiveness. Such metrics will allow research and organizations to better calibrate their efforts to improve implementation leadership and implementation climate and examine their impact on clinician behaviors including implementation citizenship. The ICBS is a very brief and practical tool that can contribute to answering these questions in implementation research.

## Additional files

Additional file 1:
**Implementation Citizenship Behavior Scale (ICBS).** This file contains the ICBS measure assessing the behaviors employees perform that exceed their expected job tasks to support the implementation of evidence-based practices (EBPs). Additional file 2:
**Implementation Citizenship Behavior Scale (ICBS) Scoring Instructions.** This file contains scoring instructions for the ICBS measure.
