# Supplementary material for: Going above and beyond for implementation: the development and validity testing of the Implementation Citizenship Behavior Scale (ICBS)
Source: Implement Sci. 2015 May 7;10:65. doi: 10.1186/s13012-015-0255-8 (PMC4465615; doi:10.1186/s13012-015-0255-8)
Supplement: Additional file 1: — Implementation Citizenship Behavior Scale (ICBS). This file contains the ICBS measure assessing the behaviors employees perform that exceed their expected job tasks to support the implementation of evidence-based practices (EBPs). [file 13012_2015_255_MOESM1_ESM.pdf]

MEASURE IN DEVELOPMENT: PLEASE DO NOT USE OR CITE WITHOUT PERMISSION

# Implementation Citizenship Behavior Scale (ICBS)

Mark G. Ehrhart
mehrhart@mail.sdsu.edu

Gregory A. Aarons
gaarons@ucsd.edu

This measure assesses the behaviors employees perform that exceed their expected job tasks to support the implementation of evidence-based practices (EBPs).

Instructions: Please indicate the frequency with which [INSERT SUBORDINATE NAME] performs each of the following behaviors.

| 0 | 1 | 2 | 3 | 4 |
|---|---|---|---|---|
| **Not at all** | **Once in a While** | **Sometimes** | **Fairly Often** | **Frequently, if not Always** |

**Helping Others**

1. Assisting others to make sure they implement evidence-based practices properly ..... 0 1 2 3 4

2. Helping teach evidence-based practice implementation procedures to new team members ........................................ 0 1 2 3 4

3. Helping others with responsibilities related to the implementation of evidence-based practices ........................................ 0 1 2 3 4

**Keeping Informed**

4. Keeping informed of changes in evidence-based practice policies and procedures .... 0 1 2 3 4

5. Keeping up with the latest news regarding evidence-based practices ........................ 0 1 2 3 4

6. Keeping up with agency communication (announcements, memos, and so on) related to evidence-based practice ........................................ 0 1 2 3 4
