# Supplementary material for: Going above and beyond for implementation: the development and validity testing of the Implementation Citizenship Behavior Scale (ICBS)
Source: Implement Sci. 2015 May 7;10:65. doi: 10.1186/s13012-015-0255-8 (PMC4465615; doi:10.1186/s13012-015-0255-8)
Supplement: Additional file 2: — Implementation Citizenship Behavior Scale (ICBS) Scoring Instructions. This file contains scoring instructions for the ICBS measure. [file 13012_2015_255_MOESM2_ESM.pdf]

MEASURE IN DEVELOPMENT: PLEASE DO NOT USE OR CITE WITHOUT PERMISSION

Implementation Citizenship Behavior Scale (ICBS) Scoring Instructions

Mark G. Ehrhart
mehrhart@mail.sdsu.edu

Gregory A. Aarons
gaarons@ucsd.edu

This measure assesses the behaviors employees perform that exceed their expected job tasks to support the implementation of evidence-based practices (EBPs).

Abbreviated Items and Scoring

Item #	Scale	Factor Loading	Alpha
	**Scale 1: Helping Others**		.93
1	Assisting others to make sure they implement EBP	.92	
2	Helping teach EBP implementation procedures	.87	
3	Helping others with responsibilities related to EBP	.86	
	**Scale 2: Keeping Informed**		.91
4	Keeping informed of changes in EBP policies	.92	
5	Keeping up with the latest news regarding EBP	.88	
6	Keeping up with agency communication related to EBP	.79	
	**ICBS Total**		.93

SCORING THE SCALES

The score for each subscale is created by computing a mean score for each set of items that load on a given subscale. For example, items 1, 2, and 3 constitute Scale 1 (Helping Others).

COMPUTING THE TOTAL SCORE

A mean of the scale scores may be computed to yield the mean score for the total ICBS.

You may contact Dr. Aarons (gaarons@ucsd.edu) for additional information or to request permission to use the measure.
